# Targeting imported malaria through social networks: a potential strategy for malaria elimination in Swaziland

**DOI:** 10.1186/1475-2875-12-219

**Published:** 2013-06-27

**Authors:** Kadiatou Koita, Joseph Novotny, Simon Kunene, Zulizile Zulu, Nyasatu Ntshalintshali, Monica Gandhi, Roland Gosling

**Affiliations:** 1 Global Health Group, University of California, San Francisco, CA, USA; 2 Clinton Health Access Initiative, Mbabane, Swaziland; 3 National Malaria Control Programme, Manzini, Swaziland; 4 Divisions of HIV/AIDS and Infectious Disease, University of California, San Francisco, CA, USA

**Keywords:** Malaria, Elimination, Importation, Swaziland, Active case detection, Social networks

## Abstract

**Background:**

Swaziland has made great progress towards its goal of malaria elimination by 2015. However, malaria importation from neighbouring high-endemic Mozambique through Swaziland’s eastern border remains a major factor that could prevent elimination from being achieved. In order to reach elimination, Swaziland must rapidly identify and treat imported malaria cases before onward transmission occurs.

**Methods:**

A nationwide formative assessment was conducted over eight weeks to determine if the imported cases of malaria identified by the Swaziland National Malaria Control Programme could be linked to broader social networks and to explore methods to access these networks.

**Results:**

Using a structured format, interviews were carried out with malaria surveillance agents (6), health providers (10), previously identified imported malaria cases (19) and people belonging to the networks identified through these interviews (25). Most imported malaria cases were Mozambicans (63%, 12/19) making a living in Swaziland and sustaining their families in Mozambique. The majority of imported cases (73%, 14/19) were labourers and self-employed contractors who travelled frequently to Mozambique to visit their families and conduct business. Social networks of imported cases with similar travel patterns were identified through these interviews. Nearly all imported cases (89%, 17/19) were willing to share contact information to enable network members to be interviewed. Interviews of network members and key informants revealed common congregation points, such as the urban market places in Manzini and Malkerns, as well as certain bus stations, where people with similar travel patterns and malaria risk behaviours could be located and tested for malaria.

**Conclusion:**

This study demonstrated that imported cases of malaria belonged to networks of people with similar travel patterns. This study may provide novel methods for screening high-risk groups of travellers using both snowball sampling and time-location sampling of networks to identify and treat additional malaria cases. Implementation of a proactive screening programme of importation networks may help Swaziland halt transmission and achieve malaria elimination by 2015.

## Background

Currently, 109 countries have succeeded in eliminating malaria [1], and 34 of the 99 countries with ongoing transmission are in the process of elimination [2]. Included in this number is Swaziland, a small country in southern Africa sharing borders with South Africa and Mozambique. The Lubombo Spatial Development Initiative (LSDI), a collaboration between Swaziland, Mozambique and South Africa for economic development, made a considerable contribution to the reduction of malaria prevalence in the region with effective treatment and vector control [3,4]. Swaziland has made striking progress in reducing its confirmed cases of malaria from 4,005 in 1999-2000 (July-June) to 369 in 2011-2012 [5] and was the first malaria programme in sub-Saharan Africa to declare an elimination goal and time frame [6].

Swaziland has introduced a strategic plan to reach its elimination goal by 2015 [7]. This includes targeted vector control using indoor residual spraying and insecticide-treated bed nets, confirmation of suspected malaria cases with a diagnostic test, immediate reporting of cases via an automated notifiable diseases tracking system, and an active surveillance system that aims to investigate all cases within 48 hours of notification [5]. Moreover, all reported cases are swiftly treated with artemisinin combination therapy; information, education and communication (IEC) campaigns target travellers to high risk areas and chemoprophylaxis is provided in local clinics [5].

It has become apparent with improved surveillance and case investigation that imported malaria, deriving almost entirely from Mozambique (greater than 90%) [7], drives malaria transmission in Swaziland. The Swaziland National Malaria Control Program (NMCP) classifies a malaria case as imported based upon the individual’s travel history within the previous two weeks and the tracing of the origin of the disease to be outside the country [8]. In the high transmission season from January to June 2012, 78% of the malaria cases were imported [5]. This importation is demonstrated most dramatically by a seasonal peak of imported malaria cases, followed a few weeks later by a rise in locally transmitted cases, as workers from Mozambique return to Swaziland in January following the Christmas and New Year holidays [9]. The potential of imported infections to lead to onward transmission is supported by a recent study showing that distance from an imported case is a predictor of risk of being a local case [9]. Because some of these imported infections may be in semi-immune adults, the infections can be carried asymptomatically and miss detection by the passive surveillance system [10], leading to infection of local mosquitoes [11]. In order to achieve elimination in Swaziland, it is critical for the National Malaria Control Programme (NMCP) to seek out these imported infections and prevent them from causing onward transmission.

Screening of people crossing a border without regard to symptoms is a potential method for identifying imported cases before onward transmission occurs, but in low transmission areas the chance of detecting cases is very low, unlikely to be cost-effective [12], and unpopular due to delays in waiting for test results. In January 2012, the Swaziland NMCP conducted a mass border screening and found a very small number of malaria positive over a large number of individuals tested (personal communication). Moreover, border screening can be even more challenging across land borders, especially in sub-Saharan Africa where most borders are porous. It may be possible to screen certain high-risk groups crossing into Swaziland but these high-risk groups have not yet been identified in this setting [7]. One possible method to identify high-risk groups who may import malaria is to explore the social networks of previously identified imported cases. These networks could be reached through methods of chain referral, commonly called snowball sampling—selected individuals are asked to provide a limited number of contacts after they accept to participate in the study; their contacts are then asked to participate in the study and give a limited number of contacts that are also approached and asked to participate and the chain can continue until the desired sample is reached [13]—or networks could be reached through time-location sampling (TLS), where social networks are accessed at specific locations at certain times. Once identified, the high-risk groups can be targeted with suitable interventions to reduce malaria importation rates and prevent onward transmission.

The study aims to describe the cross-border movements of imported cases of malaria, to determine if those individuals importing malaria are part of broader social networks, and to identify potential methods within the context of Swaziland to screen those networks for malaria infection. The ultimate aim of this study is to inform interventions to reduce importation in Swaziland, thereby reducing local transmission, and achieving elimination.

## Methods

### Study design

A qualitative formative assessment of social networks

### Study site: Swaziland

Swaziland is a small landlocked country located in southern Africa, between Mozambique and South Africa, with an estimated population of 1.2 million [14], and a current national malaria prevalence of 0.2% [15]. The study was conducted nationwide.

### Study population

The study population was comprised of the providers from health facilities that diagnosed imported cases, malaria surveillance officers, and cases of malaria classified by the NMCP as imported (index cases) within the previous three months, along with contacts (i.e. networks) of the index cases.

### Sample size

As this was a qualitative pilot, no formal calculation was made for sample size. The goal of the study was to interview 10 health care providers from clinics that identified imported malaria cases, six of the 12 surveillance agents from the NMCP, a maximum of 50 imported malaria cases, and a maximum of 35 network members and key informants.

### Study procedures

A series of preliminary interviews were conducted to inform later queries and discussion points for index case interviews. These included interviews with health facilities providers and surveillance agents to hear their perspectives on the importation of malaria and the contexts in which cases were imported. The names of the six surveillance agents were randomly picked from a hat containing the names of all the agents written on pieces of paper. The agents were asked about their work, the imported cases of malaria they had observed, as well as asked to provide potential solutions to malaria importation in Swaziland. Facilities were also randomly chosen using the same method and names of providers were randomly chosen from the names of providers who reported malaria cases over the previous three months from the chosen facilities. One provider was selected per facility. Providers were asked about their work, malaria management, imported cases of malaria they had seen, and about potential solutions to malaria importation.

Imported malaria cases from the preceding three months were selected from the NMCP Malaria Surveillance Database System and names and contact details of the imported cases were extracted. Imported malaria cases were selected as potential “seeds” of high-risk networks for two reasons. Firstly, the case was determined to be at-risk for importing malaria by definition because the individual did import malaria, and secondly, this sampling method could be easily utilized by the NMCP.

Each imported case was telephoned by a surveillance agent and was given a brief description of the study. If the individual agreed to participate in the study, he or she was asked where and when would be the best place and time for an interview. An appointment was made with the participant at his/her preferred location. Using a semi-structured interview guide the cases were asked about their demographic information, their travel patterns and reasons for travel, the contact details of people they knew to have similar travel patterns that might have put them at risk of getting malaria (i.e. networks), and the best way to find those contacts. They were also asked about their thoughts on the study, the attitudes their networks would have towards the study and malaria testing.

The contact details of the network contacts were collected during the interviews of the imported cases. The networks were telephoned and given a brief description of the study and were asked if they would participate. They were also interviewed at their preferred locations during their preferred times. All the participants were read an informed consent in the language they understood. In addition to the questions asked of the index cases, additional questions assessed their willingness to be tested for malaria. The interviews were conducted by a study investigator assisted by an interpreter whenever needed and were tape-recorded with the participants’ consent. All recorded interviews were transcribed; interviews in SiSwati were translated into English with the help of translators fluent in both English and SiSwati. All interviews were then coded and analysed using Microsoft Excel for recurrent themes.

### Ethical considerations

The study was approved by the University of California, San Francisco Committee on Human Research (CHR) and the Swaziland Ministry of Health Science and Ethics Committee.

## Results

During the eight weeks of the pilot study (April-May 2012), a total of 60 interviews were conducted: 10 health care providers, 6 surveillance agents, 19 index cases and 25 network contacts. No participant refused to be interviewed. Table 1 gives a brief description of the study population.

**Table 1:** Study population

**Study participants**	**Male**	**Female**	**Total**
**Surveillance agents**	4	2	6
**Health care providers**	2	8	10
**Index (imported cases)**			
Mozambicans	9	5	14
Swazis	4	1	5
**Networks/key informants**			
Mozambicans	17	4	21
Swazis	3	1	4
**Total**	39	21	**60**

### Findings from the interviews of the surveillance agents and health care providers

Most of the NMCP surveillance agents expressed the need to solve the importation problem because their experience has shown them that travellers do not often use chemoprophylaxis, so the agents were concerned travellers would continue to contract and import malaria, putting the Swazi population at risk. Agents mentioned challenges related to their everyday work. They reported language barrier as a challenge. According to the agents, most of the malaria imported cases in their catchment areas are Mozambicans. Some of them do not speak the local languages common in Swaziland and the agents do not speak Mozambican languages. Additionally, the agents reported limited resources as a hindrance to a successful case investigation. They reported that because most of the imported cases are mobile, working during the day, it is hard for them to find them at one place, and because the agents have limited mobile phone airtime to work with, they cannot call the cases to localize them prior to visiting. Furthermore, poor documentation of information necessary to follow up a case was another challenge. In some cases it was the reporting itself. According to the agents, some health providers just don’t like to report. The failure to report a case in a timely manner can cause a delay in the investigation, and raise some resentment from the agents as some revealed during the interviews:

“*You know, people just don’t want to report. I don’t know if it’s a culture of ours. There are some people who cannot recall what they have done. Sometimes they do it perfectly their job. The client goes home having been treated everything goes well. But the problem where have you written what you’ve done? You’d find that there is nothing. You’d find gaps on our reporting tools.”*

This issue of reporting was later confirmed by one provider only who complained it is a waste of time and duplicate of effort to report a case at the same time record information for the surveillance agents’ investigation.

Agents believed that the individuals who were importing malaria (mostly Mozambicans, based on their experience) knew each other and knew others with similar travel patterns that could similarly put them at risk of contracting malaria.

“… Even if the cases are Swazis you’d find that they are females and married to Mozambicans. You would find that she goes to visit her husband’s family. That’s how you’d find Swazis. But mostly, in a scale of 10 you’d find that nine are Mozambicans.”

“Definitely, they know each other. Most of our imported cases are Mozambicans who are residing here in Swaziland. They are living here, so they go home month-end or anytime when they feel like going to Mozambique for whatever business they go.”

The health care providers were aware of the activities of the NMCP and support them in diagnosing, treating, and reporting cases of malaria. When asked about the profile of a typical imported malaria case, all the providers had close answers on the similarity of their provenance, their professions, and the frequencies and reasons for their travels. The providers suggested some places and days where and when those individuals could be found.

“Most of them are staying here and they are Mozambicans and they go to Mozambique every month end those are the type of people that we see. They are road constructors we have a number of them, cane cutters, general labourers, hawkers.

“You will find them at the Manzini market where they sell clothes on Wednesday and Thursday. There is an office where they are all controlled. Those women meet regularly at that office. You can go to that office and talk to them during their mass meeting.”

With regard to importation prevention, some of the providers referred to health education through media campaigns, as well as screening at border gates. Some even proposed a law that will require travellers to carry a certificate, like the one documenting yellow fever vaccination, showing that they are malaria free and are taking chemoprophylaxis.

“I think we would need to make a law that requires visitors to have a certificate of being malaria free before coming to Swaziland, just like Yellow Fever Certificate.”

## Findings from imported cases’ interviews

### Imported cases profile

From the interviews, it was found that 14 out of the 19 imported cases were Mozambican nationals. The median age was 33 and the range was two to 53 years. The mother of the two-year old child was interviewed in the child’s place. The majority of the participants were males (13/19), mainly labour workers in either construction or farming. The females were mostly traders and street vendors of fruits and vegetables. The bulk of participants were married and their families remained in Mozambique. The Swazi participants were mostly composed of males (4/5) conducting business between Swaziland and Mozambique, or women (1/5) married or living with a Mozambican.

### Travel habits

**Figure 1:**
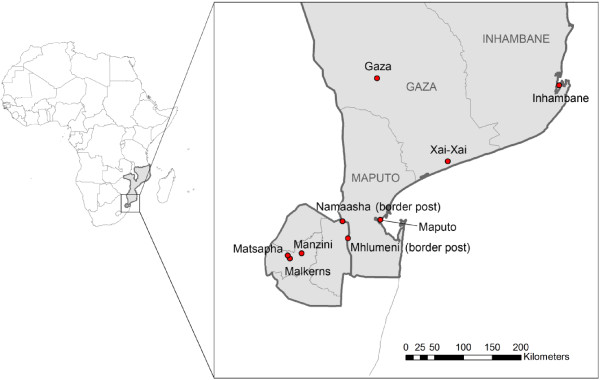
Mozambique and Swaziland maps showing the cities concerned.

The participants reported frequent travel to Mozambique. The two borders gates (Lomahasha and Mhlumeni, see Figure 1) were used in equal proportions. All the participants, but one, reported crossing the border formally. They travelled mostly in groups (consisting of other family members or friends). The frequencies of their travel varied from once a year to once a week. When asked about how often they travelled to Mozambique, the participants answered very similarly:

“My home is there so I go there frequently, maybe three times a month.”

“I usually go every month end, or after two months.”

“The kind of business requires that I go there and return the same day. Five or six times a month.”

Because their families stay in Mozambique, Mozambican participants reported a visit to home as the main reason for their frequent travel. Almost all of them travel during the Swaziland festive season (from late December to early January) to spend the Christmas and New Year holidays with their families. Many of them are working in Swaziland, but their wives and children are left in Mozambique. They pay regular visits to check on their wives and children and to provide them with money. As this participant reported:

“I go there (Mozambique) to check on my family. I have to support my children, so whenever I have some money I go there. Also my parents are there, I go from time to time to see them.”

Another theme that came out during the interviews of the Mozambican nationals was their need to travel outside Swaziland every 30 days to be eligible for re-entry in the country. They reported that they must cross the border and spend at least three days in Mozambique before coming back to Swaziland to be in compliance with legal border procedures.

Among the Swazi participants, the reasons for travelling to Mozambique were business and sometimes vacations. However, the interviews additionally revealed that the bulk of the Swazi participants went to Mozambique to seek traditional healing. A considerable number of participants (Swazis and Mozambicans) stated that Mozambique is renowned for its traditional healers. Mozambicans living in Swaziland go back to their home country to access traditional healing services and Swazis traditional healers go to Mozambique to learn from the local healers or to seek treatment.

“I don’t usually go there except when people ask me to help take them to seek treatment. I have epileptic children so there is a woman that treats them there. I had gone to see her.”

What I’ve learnt is that usually most of the cases come from the same area, which is if I’m not mistaken it is Inhambane where people go there for different purposes like collecting “muti” (traditional medicine), you know Inhambane is popular for having good traditional healers.

### Existence of traveller networks

All the index cases reported knowing somebody who travelled frequently to Mozambique and who might have been at risk for malaria infection. The contact was usually a relative, friend, co-worker, neighbour, or acquaintance from the church. Their answers were similar to the statements below:

“Yes there are many of them. I know some other people who are working here in a private garage in Matsapha, who normally travel to Mozambique. I am in constant contact with them. It is a group of men who are working here, and other women who work here. So I can contact them and inform you through the numbers I received today, if there is a group leaving for Mozambique, or a group leaving Mozambique to here.”

“There’s one who works here who has had malaria. I know a number of them and I have their numbers.”

The individuals within networks were then approached, interviewed, and asked for information on their contacts. All the contacts had mobile phones and were willing to participate in the study when they were in Swaziland. There were some cases when phones were switched off during the individual’s travel or residence in Mozambique at the time of the calls, therefore, could not be reached. The individuals referred were contacted usually the same day or the following and were interviewed within average of three days and a maximum of a week based on the investigators’ schedule and plan, which was to conduct interviews by area. Sometimes the contacts were interviewed the same day if they were close by. All contacts were willing to be tested for malaria when asked. They were comfortable with the idea of being interviewed and tested at their work places. Though, the testing was not part of this study since it was a formative assessment to determine the possibility of testing.

## Findings from the network contacts’ interviews

### Network profiles

**Figure 2:**
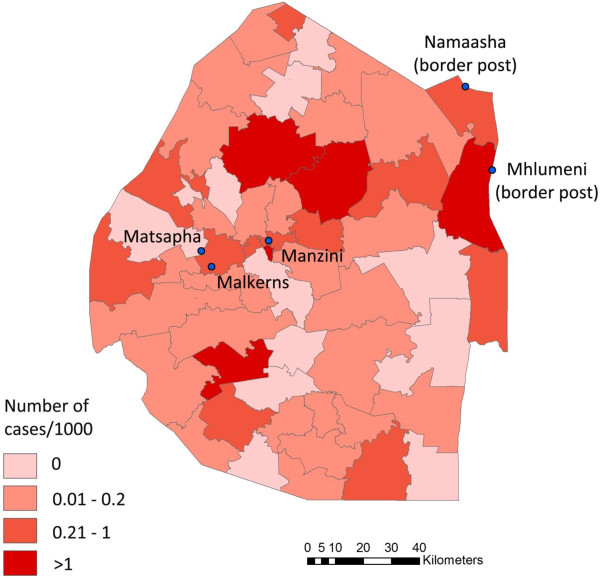
Cities of interest overlying malaria incidence by Tinkhundla (second administrative level) over the period of July 2011 - June 2012.

Of the 25 network contacts interviewed, 21 were Mozambicans. They all lived in Swaziland either conducting small businesses or working in skilled labour. Only 20% of the participants were women. The bulk of the participants were between the age of 25 and 40. The interviews revealed that the majority of the network contacts were concentrated in Malkerns, a town located at 20 km from Manzini (see Figure 2). Many are self-employed in metalwork and selling their products at local market places. A number were on contracts working as bricklayers, house constructors or carpenters. Others were employed on farms caring for chickens, livestock and/or sugar cane plantations. The women were mostly selling fruits and vegetables at local market places and busy intersections. As some network participants stated:

“Mozambicans work in all the sectors. They are in the agricultural sector, kitchens, factories, industries.”

“They are hawkers, Swazis who go to Mozambique to buy clothes and selling them here…”

### Travel habits

All the network contacts interviewed claimed to be travelling between Swaziland and Mozambique. While some contacts (6/25) had not travelled within the two months preceding the interview, other stated they had come back in the week of the interview. The frequencies varied between two to five times a year to two to three times a month and crossed the borders formally using public transportation the majority of the time. Only one participant reported travelling once a year. The majority of the participants stated they travelled with friends, family, or co-workers. The reasons for travel were similar to those of the imported cases. The areas most visited during their travel were Maputo and Inhambane cities (see Figure 1). Maputo is thought to have lower malaria prevalence than the central and northern Mozambique based on the 2007 Malaria Indicator Survey. A few participants reported visiting Xai-Xai (see Figure 1), in Gaza province where malaria prevalence was about 10% in 2010 [16]. The two border gates seemed to be equally used and none of the participants reported having crossed the border informally. There was no difference in answers between ages.

### Attitude toward malaria and prevention methods used

Some individuals in the networks reported using a prevention method. They all agreed to be tested for malaria when asked by the interviewer. The majority of them (16/25) reported not having any episode of malaria within the month preceding the interview. Four individuals reported never having had malaria, and five individuals cited that they self medicated if they had a fever or headache.

“I did not go to hospital but to a chemist.”

“These days I don’t get sick besides having slight headache and drink some panadol (painkiller).”

“I don’t know if it was malaria but I had a headache and fever last month. I usually have the headaches. Almost every two weeks. I always keep painkillers in the house. I also had it yesterday.”

### Screening methods identified

The individuals within the networks of imported cases were identified through a chain of referral and contacted through telephone numbers collected from the imported cases. The network members were successfully contacted and stated a willingness to be tested for malaria.

A few important key informants were identified among the network contacts, including a leader from the Mozambican Association in Swaziland and other local businessmen, who claimed to know many people who travel frequently to Mozambique (both Mozambicans and Swazis). These key informants claimed to have the ability to refer travellers, and indicated willingness to organize frequent travellers in future studies, allowing for the possibility for a TLS method for a future intervention.

## Discussion

This study has shown that patients identified through passive surveillance and classified as imported cases by the Swaziland NMCP have networks of contacts who can easily be reached and screened for malaria infection. This preliminary study has implications for the mitigation of imported malaria in Swaziland. Individuals with similar high-risk travel patterns to those of imported malaria cases in Swaziland were able to be contacted and expressed their willingness to be tested for malaria. Key informants among the network contacts were identified and the locations were given where members of the Mozambican community could be accessed. These findings indicate the possibility of using new social network strategies within an active surveillance programme to reduce the risk of onward local transmission caused by imported malaria in elimination settings.

This study found that networks of high-risk travellers can be accessed. All of the index cases reported knowing at least someone who travelled frequently between Swaziland and Mozambique who might be at risk for carrying malaria parasites. Networks were made up mostly of family members and friends, co-workers, or acquaintances from the church. Networks were mostly comprised of Mozambicans living and working in Swaziland while their families remained in Mozambique. The majority were skilled workers in construction or farms or conducting small businesses as metal workers, traders, shoemakers and street vendors of fruits and vegetables. These findings complement the results of Tatem and Smith [17] who found that most of the population movements between malaria-endemic countries and countries working on elimination is related to trade or family visits; this population movement can contribute to the importation of parasites.

The majority of the network contacts in this particular setting travel to Maputo and Inhambane in Mozambique where they have their homes. This aligns with findings of the NMCP active surveillance programme demonstrating that the majority of imported cases come from these two areas (unpublished data). Many of the network contacts reported travelling to those two areas, showing the likelihood that those networks contain infected individuals. Since these contacts reside in receptive areas in Swaziland (see Figure 2), they could lead to onward transmission. Identifying where individuals have been traveling and linking this information with transmission maps might help to further target screening. Furthermore, cases now residing in more receptive areas in Swazi, which can be established from recent risk mapping work, would help to further prioritize networks.

The interviews determined that most of the travellers do not use malaria chemoprophylaxis prior to travelling. Additionally, some participants reported having auto-medicated themselves with painkillers when they had malaria symptoms. This could interfere with malaria detection by passive surveillance systems and can facilitate the onward transmission of malaria. It has been reported that the incubation time for some *plasmodium* species can last up to four months [18]. Since the majority of the imported malaria cases come from Mozambique where *Plasmodium vivax* and *Plasmodiun malariae,* which are known to have longer incubation time, are present [19] there is a possibility that individuals infected with these species may transmit the infection to others even months after their travel.

The study showed that network contacts can be identified through chains of referral and be contacted through telephone numbers collected from the index cases. The study used traditional snowball sampling methods whereby the index case interviewed gives the names and contact details of his/her contacts. This method of sampling has been shown to be effective in HIV studies in contacting hard-to-reach populations, such as men who have sex with men and sex workers [20]. Snowball sampling has also been used in studies of non-heterosexual women [21], and recreational drug users [22]. This method may be an effective strategy in the future within active surveillance systems to rapidly identify imported cases of malaria in Swaziland.

Key informants were identified among the networks, including leaders from the Mozambican Association in Swaziland and other local businessmen who claimed to know many people who travel frequently to Mozambique (Mozambicans and Swazis) and whom they can refer if needed. These key informants claimed to have the ability to organize and assemble at a specific place and time those who travel frequently to Mozambique, opening the possibility for a TLS method in future intervention studies.

This study demonstrated the feasibility of identifying traveller networks for eventual screening and testing for malaria in order to reduce the impact of malaria importation in Swaziland. Once the travellers at risk are identified, special measures can be taken to reduce the risk of onward transmission resulting from importation, including targeted vector control among these groups, continuous screening and treatment of high-risk networks, and the information and education campaigns promoting person protection and treatment seeking behaviour.

### Limitations

This study cannot determine the risk of malaria among the network contacts since no testing was performed and the study period was short. The findings might be different if the study was done at a different time, for example during the peak of imported cases in January. It is possible that the participants might not really acknowledge where they have been or how they crossed the border, even though there was no indication that they did not. Because network contacts were not tested for malaria in this study, the prevalence of malaria among these groups could not be determined and the research could not ascertain how far to continue sampling. The further sampling would have cost and time implications for surveillance agents if adopted as a strategy. Further studies which include testing of individuals are planned and should help to resolve these issues. Since no study has yet evaluated traveller networks associated with malaria importation into Swaziland to date, there is no reference for comparable results.

## Conclusion

Imported malaria is a problem in countries in the process of eliminating malaria or preventing re-introduction. Strategies to improve the detection of imported cases of malaria are needed. This study showed that imported cases of malaria identified by the Swaziland NMCP are part of broader social networks. These networks are composed mostly of family members, relatives, friends and co-workers. Contacts are generally Mozambicans living and working in Swaziland, travelling frequently to Mozambique to visit their families or conduct business. In a few cases, the contacts were Swazis travelling to Mozambique for business purposes. They were identified through methods of chain referral (snowball sampling). High-risk networks of travellers can likely be organized at a specific place at specific times using TLS methods in the future. They can be contacted through telephone calls and be tested for malaria. This study shows that it is possible for malaria control programmes to use social networking methods to identify and access high-risk traveller groups and to potentially deliver interventions. What remains unknown is the actual risk of malaria infection within these networks. Further studies to estimate these risks are needed to establish the utility of malaria network sampling as a new tool for malaria control and elimination.

## Competing interests

The authors declare that they have no competing interests.

## Authors’ contributions

KK, MG, JN, and RG designed the study proposal; KK conducted and analysed the interviews, and interpreted the results; KK, JN, SK, ZZ, and NN supported and supervised data collection; KK, JN, NN, SK, ZZ, MG, and RG wrote the manuscript. All authors read and approved the manuscript.
